# The Percentage of Signet Ring Cells Is Inversely Related to Aggressive Behavior and Poor Prognosis in Mixed-Type Gastric Cancer

**DOI:** 10.3389/fonc.2022.897218

**Published:** 2022-05-26

**Authors:** Luigi Marano, Maria Raffaella Ambrosio, Luca Resca, Ludovico Carbone, Osvaldo Carpineto Samorani, Roberto Petrioli, Vinno Savelli, Maurizio Costantini, Lara Malaspina, Karol Polom, Ivano Biviano, Daniele Marrelli, Franco Roviello

**Affiliations:** ^1^ Department of Medicine, Surgery and Neurosciences, Unit of General Surgery and Surgical Oncology, University of Siena, Siena, Italy; ^2^ Pathology Unit, University of Siena, Siena, Italy; ^3^ Pathology Unit, Azienda USL Toscana Nord-Ovest, Pisa, Italy; ^4^ Department of Medicine, Surgery and Neurosciences, Unit of Medical Oncology, University of Siena, Siena, Italy; ^5^ Department of Surgical Oncology, Medical University of Gdansk, Gdansk, Poland; ^6^ Gastroenterology and Operative Endoscopy Unit, AOU Senese, Siena, Italy

**Keywords:** mixed-type gastric cancer, signet ring cell, prognosis, histology, poorly cohesive

## Abstract

**Background and Objectives:**

Only recently the percentage of signet ring cells (SRCs) in gastric cancer (GC) has been proposed as an independent predictor of survival. High amounts of SRCs have been related to lower recurrence and mortality rates, better prognosis, and favorable clinicopathological features in a poorly cohesive histotype. It is not known what the effect of SRC percentage in mixed-type GC is. We investigate the role of SRCs as a prognostic marker in mixed-histotype GC.

**Methods:**

A retrospective analysis was performed through a prospectively maintained database of patients with diagnosed “mixed-type” gastric carcinoma, defined according to 2019 WHO classification. These patients underwent surgery between 1995 and 2016, and their tissue samples were stored in a tissue bank. All slides were analyzed, and patients were divided into three groups according to the percentage of SRCs: “Group 1” (displaying ≤10% of SRCs), “Group 2” (displaying <90% but >10% of SRCs), and “Group 3” (displaying ≥90% of SRCs). We compared clinical and pathological features as well as prognostic factors between the different groups.

**Results:**

Among 164 enrolled patients, 68.9% were male and 31.1% were female (*p* = 0.612). The mean (±SD) age at diagnosis was 71.4 ± 9.6 years. Ninety-eight (59.7%) patients were classified as “Group 1”, 66 (40.3%) as “Group 2”, and none as “Group 3”. Five-year overall survival was remarkably higher in Group 2 (73.8%) in comparison to Group 1 (35.4%), *p* < 0.001. Mortality risk was three times higher in patients with ≤10% SRC pattern compared to those with >10% [HR 2.70 (95% CI 1.72–4.24)]. After adjusting according to potential confounding factors, SRC percentage was still an independent predictor of survival.

**Conclusions:**

The proportion of SRCs is inversely related to aggressive behavior and poor prognosis in mixed-type GCs, highlighting the role of SRC amount as an independent predictor of survival.

## Introduction

Gastric cancer (GC) is the fifth most diagnosed malignancy worldwide with over 1 million estimated new cases annually (1). Due to its intratumoral and intertumoral huge heterogeneity, several classifications have been proposed to categorize different morphological subtypes of GC. The most commonly used classifications are those published by the Japanese Gastric Cancer Association (JGCA) (2), the World Health Organization (WHO) (3), and Lauren (4). However, a categorization into a few macro groups is burdened by excessive internal heterogeneity, resulting in conflicting evidence about the ability of the histopathological phenotype to predict patient prognosis or response to therapy. In 2019, the European Chapter of the International Gastric Cancer Association (IGCA) proposed the adoption of the recent fifth edition of the WHO classification for each newly diagnosed GC (5, 6). The latest classification recognized five main histological subtypes of GC: tubular, papillary, mucinous, poorly cohesive, and mixed adenocarcinomas. The latter account for 6%–27% of all GCs and are characterized by the coexistence of two or more distinct histological components: glandular (tubular/papillary) and signet ring cell (SRC)/poorly cohesive. Recently, the percentage of SRC was proposed as an independent prognostic factor of cancer-related survival in a poorly cohesive histotype, and high amounts of SRC were related to lower recurrence and mortality rates, better prognosis, and most advantageous clinicopathological features (6–8). Surprisingly, clinicopathological and prognostic aspects of mixed histotype have not been deeply investigated. Available data suggest that patients with mixed adenocarcinomas had a poorer prognosis (9, 10) and higher incidence of lymph node metastasis than those with only one component. Furthermore, to the best of our knowledge, no previous research has studied the connection between the amount of SRCs and biological as well as prognostic differences of mixed GC.

We conducted this study to compare the clinicopathological features and the prognostic differences of mixed GC according to the percentage of SRCs.

## Patients and Methods

A retrospective analysis was performed through a prospectively maintained database of adult patients from the Division of Surgical Oncology at the University of Siena, Italy. These patients all underwent upfront surgery for GC between 1995 and 2016, and their tissue samples were available for research at an institutionally approved tissue bank. Only patients with confirmed “mixed” gastric carcinoma, according to the last WHO classification (3), submitted to surgical treatment with curative purpose, were recruited for the analysis. All enrolled patients were then reclassified into the three groups according to the percentage of cells with signet ring features as proposed by the European Chapter of IGCA (6): Group 1 (presenting ≤10% of SRCs), Group 2 (presenting <90% but >10% of SRCs), and Group 3 (presenting ≥90% of SRCs).

Electronic medical records and pathological reports were thoroughly analyzed to acquire information related to age, sex, tumor size, location, depth of invasion, perineural/lymphovascular invasion, lymph node metastases, and TNM (8th edition) according to the American Joint Committee on Cancer Staging Manual (11).

### Treatment Strategy and Clinical Information

Patients underwent similar management, according to the recommendations of the multidisciplinary team. Serosal invasion and minimal peritoneal disease were evaluated utilizing an optimized dedicated protocol through contrast-enhanced thoracoabdominal computed tomography (CT), with the help of an experienced radiologist. In selected cases, where CT scan results were doubtful and peritoneal carcinosis could not be ruled out, staging laparoscopy with a cytological examination of peritoneal lavage was also performed.

Experienced surgeons performed surgery according to treatment guidelines published by the Japanese Gastric Cancer Association (ver. 4) (12). The first-line treatment to remove the primary tumor consisted of total or subtotal gastrectomy. Combined visceral resections were conducted to remove all visible lesions, in a case-by-case evaluation. D1 lymphadenectomy was carried out in cases of early GC. When gastric wall layers deeper than muscularis propria were involved, D2 or D3 lymphadenectomy was performed.

Death within 90 days of surgery was considered as postoperative mortality. Systemic chemotherapy treatment consisting of a combination of fluoropyrimidine and platinum regimens was administered to patients after complete recovery and hospital discharge. Eventually, biological therapy was added, according to patients’ general status and multidisciplinary evaluations.

### Follow-Up

After surgery, patients were examined at set intervals for both surgical and oncological follow-up. Blood tests (including tumor biomarkers), as well as CT, were performed every 3 months for the first 2 years, every 6 months from years 3 to 5, and yearly after that date, unless otherwise requested according to clinical status.

We differentiated recurrence as loco-regional relapse, distant organ metastasis, and peritoneal dissemination. Cancer recurrence at the anastomotic site or around the surgical area was included in the loco-regional relapse group. On the other hand, liver and other extra-abdominal site metastases, and nodal metastases beyond regional nodes were classified as distant recurrences.

Tumor recurrence was evaluated through clinical findings, radiological exams, endoscopic examination, and/or tissue biopsy.

### Histopathological Assessment

Two pathologists (MA and LMal), experts in the gastro-intestinal pathology field, blindly reviewed hematoxylin and eosin (H&E) slides obtained from the patients enrolled in the study. All the cases were re-classified according to the last WHO classification (3) and IGCA classification (6). The mixed histotype was also confirmed using E-cadherin immunohistochemistry, which should be restricted to the SRC/poorly cohesive component. Moreover, in the poorly cohesive component, the percentage of SRC in respect to the whole morphology was assessed.

### Study Endpoints and Definition

The main goal of this study was to compare the overall survival (OS), defined as the interval time from the date of intervention until the death of any cause or last available contact, in the three groups. The recurrence-free survival (RFS) was also investigated, from the day of intervention until loco-regional or distant recurrence evidence. The secondary aims were a comparative analysis of clinicopathological features, mortality, morbidity, and prognostic factors for survival.

### Statistical Analysis

The data we gathered were analyzed and found to be normally distributed using the Shapiro–Wilk *W* test. These data are presented as means ± standard deviation (SD). An unpaired *t*-test, or Pearson’s chi-squared (*χ* ^2^), was used appropriately to assess differences among groups. We used the reverse Kaplan–Meier method to estimate the median follow-up time (13). OS and RFS analyses were carried out between Group 1, Group 2, and Group 3 GC patients with curative resection with the use of the Kaplan–Meier estimation method and compared using the log-rank test. Hazard ratios (HRs) were calculated using the Cox proportional hazards regression with 95% confidence intervals to account for risk factors. The endpoint is defined as death from cancer. The model included the following covariates: age (<60 years and ≥60 years), lymph node metastasis (negative and positive), lymphovascular invasion (negative and positive), perineural invasion (negative and positive), EGC/AGC (early gastric cancer and advanced gastric cancer), TNM stage (1–2 and 3–4), and SRC proportion (Group 1 and Group 2). Variables were selected using the stepwise forward procedure, and *p*-values < 0.05 were considered significant for their inclusion. *p*-values > 0.1 were considered significant for their removal. At each step, the variable with the highest statistic score was added by the model. Global *χ* ^2^ changes from each previous step and residual *χ* ^2^ were calculated at each step. Beta coefficients, HRs, and their 95% confidence interval were estimated for each significant variable and compared with its reference category. Missing items were excluded or analyzed in a separate group if exceeding 5%. A *p*-value < 0.05 was considered statistically significant, and all statistical analyses were performed using the SPSS version 26.0 software package for Mac (IBM Corp., Chicago, IL, USA).

The present study complies with STROBE (Strengthening the Reporting of Observational Studies in Epidemiology) guidelines (**Supplementary Table 1**) (14).

## Results

### Clinicopathological and Surgical Characteristics of Patients With Poorly Cohesive Carcinoma

**Figure 1:**
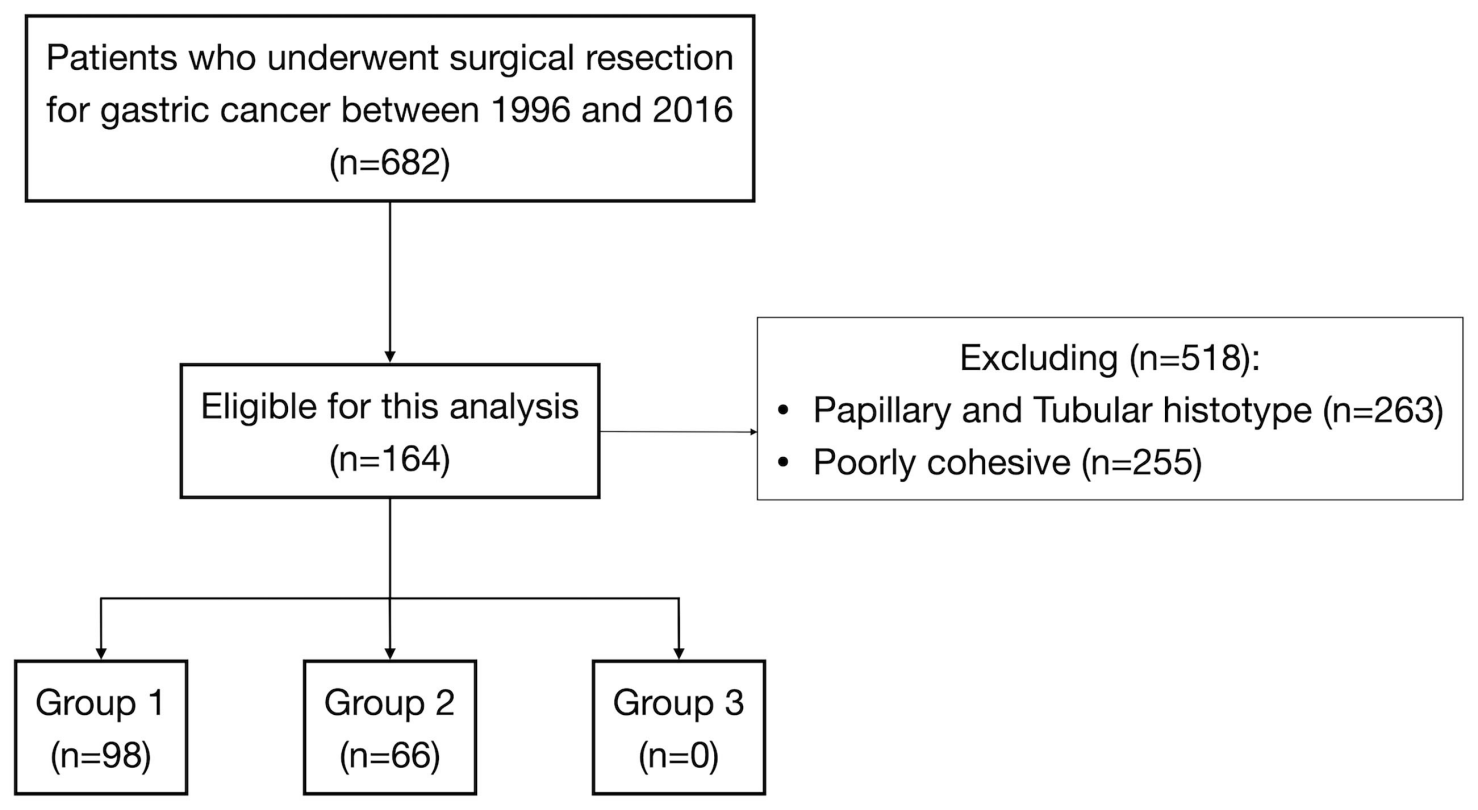
Flow diagram of patient selection among 682 patients submitted to surgical resection for gastric cancer.

Of 682 GC patients, 263 (38.6%) were confirmed as papillary, tubular, or mucinous histotype. Two hundred fifty-five patients (37.4%) were diagnosed as “poorly cohesive”, while 164 (24%) were confirmed as mixed histotype according to the last WHO classification (3). These patients were included in our study, and the specimens of all mixed cancers were available in an institutionally approved tissue bank (**Figure 1**). The proportion of female was 31.1% (*n* = 51) and the mean (±SD) age at diagnosis was 71.4 ± 9.6 years. Twenty-five cases (15.2%) were diagnosed with EGC and 139 (84.8%) were diagnosed with AGC; in most cases, the lesions started from the distal part of the stomach (50%). **Table 1** summarizes the clinicopathological characteristics of 164 patients with mixed-type gastric carcinoma. In all cases, surgery was meant to be curative. Total gastrectomy was carried out in 33.5% (*n* = 55), while subtotal gastrectomy was performed in 66.5% (*n* = 109). D2 was the most common lymphadenectomy, while D1 was executed in 26.2% (*n* = 43) and D3 was executed in 8.5% (*n* = 14) of patients. At the histopathological evaluation, most cancers were pT3–4 (61%, *n* = 100) and pN+ (52.4%, *n* = 86), and with lymphovascular invasion (47.9%, *n* = 58) and neural invasion (17.6%, *n* = 21) (**Table 2**). No postoperative deaths were registered. After surgery, 95 patients (57.9%) completed systemic chemotherapy, while the remaining were not able to start the treatment due to postoperative complications that delayed discharge and recovery.

**Table 1:** Clinicopathological characteristics of patients with mixed-type gastric cancer according to SRC percentage.

Variables	All patients (*n* = 164)	SRC ≤ 10 (*n* = 98)	SRC > 10 (*n* = 66)	*p*-value
Age (years) mean ± SD	71.4 ± 9.6	71.2 ± 9.4	71.7 ± 9.8	0.763
Gender				0.612
Female	51 (31.1)	29 (29.6)	22 (33.3)	
Male	113 (68.9)	69 (70.4)	44 (66.7)	
Location of tumor				0.499
Upper third	22 (23.4)	14 (14.3)	8 (12.1)	
Middle third	52 (31.7)	29 (29.6)	23 (34.8)	
Lower third	82 (50)	50 (51)	32 (48.5)	
Diffuse	8 (4.8)	5 (5.1)	3 (4.5)	
EGC/AGC				**0.000**
Early Gastric Cancer	25 (15.2)	5 (5.1)	20 (30.3)	
Advanced Gastric Cancer	139 (84.8)	93 (94.9)	46 (69.7)	
Adjacent organs infiltration				0.261
No	149 (90.9)	87 (88.8)	62 (93.9)	
Yes	15 (9.1)	11 (11.2)	4 (6.1)	
Grading				**0.001**
G1	19 (11.6)	5 (5.1)	14 (21.2)	
G2	22 (56.1)	53 (54.1)	39 (59.1)	
G3	45 (27.4)	34 (34.7)	11 (16.7)	
Tumor size (mm), mean ± SD	50.9 ± 26.4	56.7 ± 26.4	42.5 ± 24.1	**0.001**
Staging-T				**0.000**
T1–2	64 (39)	21 (21.4)	43 (65.2)	
T3–4	100 (61)	77 (78.6)	23 (34.8)	
Staging-N				**0.000**
N0	78 (47.6)	26 (26.5)	52 (78.8)	
N+	86 (52.4)	72 (73.5)	14 (21.2)	
Staging-M				0.786
M0	153 (93.3)	91 (92.9)	62 (93.9)	
M1	11 (6.7)	7 (7.1)	4 (6.1)	
AJCC TNM Stage 8th ed.				**0.000**
Ia	23 (14)	4 (4.1)	19 (28.8)	
Ib	27 (16.5)	9 (9.2)	18 (27.3)	
IIa	26 (15.9)	10 (10.2)	16 (24.2)	
IIb	15 (9.1)	11 (11.2)	4 (6.1)	
IIIa	14 (8.5)	12 (12.2)	2 (3)	
IIIb	14 (8.5)	13 (13.3)	1 (1.5)	
IIIc	27 (16.5)	25 (25.5)	2 (3)	
IV	18 (11)	14 (14.3)	4 (6.1)

Data are expressed as n (%) unless otherwise specified and percentages are given according to number of patients per line after exclusion of patients with potential missing data. SD, standard deviation; SRC, signet ring cell; EGC, early gastric cancer; AGC, advanced gastric cancer; AJCC, American joint committee on cancer. P values in bold are statistically significant.

**Table 2:** Surgical characteristics of patients with mixed-type gastric cancer according to SRC percentage.

Variables	All patients (*n* = 164)	SRC ≤ 10 (*n* = 98)	SRC > 10 (*n* = 66)	*p*-value
Lymphovascular invasion				**0.001**
Negative	63 (38.4)	26 (26.5)	37 (56.1)	
Positive	58 (35.4)	44 (44.9)	14 (21.2)	
Perineural invasion				0.155
Negative	98 (59.8)	52 (53.1)	46 (69.7)	
Positive	21 (12.8)	16 (16.3)	5 (7.6)	
Surgical procedure				**0.085**
Total gastrectomy	55 (33.5)	36 (36.7)	19 (28.8)	
Subtotal gastrectomy	109 (66.5)	62 (63.2)	47 (71.2)	
Lymphadenectomy				0.392
D1	43 (26.2)	27 (27.6)	16 (24.2)	
D2	107 (65.2)	65 (66.3)	42 (63.6)	
D3	14 (8.5)	6 (6.1)	8 (12.1)	
Resection of other organs				0.440
No	148 (90.2)	87 (88.8)	61 (92.4)	
Yes	16 (9.8)	11 (11.2)	5 (7.6)	
Radicality				**0.006**
R0	123 (75)	65 (66.3)	58 (87.9)	
R1	20 (12.2)	17 (17.3)	3 (4.5)	
R2	21 (12.8)	16 (16.3)	5 (7.6)	
Proximal margin infiltration				0.217
Negative	147 (98.7)	83 (97.4)	63 (100)	
Positive	2 (1.3)	2 (2.4)	0	
Distal margin infiltration				0.192
Negative	147 (96.1)	83 (94.3)	64 (98.5)	
Positive	6 (3.9)	5 (5.7)	1 (1.5)	

Data are expressed as n (%) unless otherwise specified and percentages are given according to number of patients per line after exclusion of patients with potential missing data. SD, standard deviation; SRC, signet ring cell. P values in bold are statistically significant.

### Clinicopathological and Surgical Characteristics of Patients Grouped by Proportion of SRC

**Figure 2:**
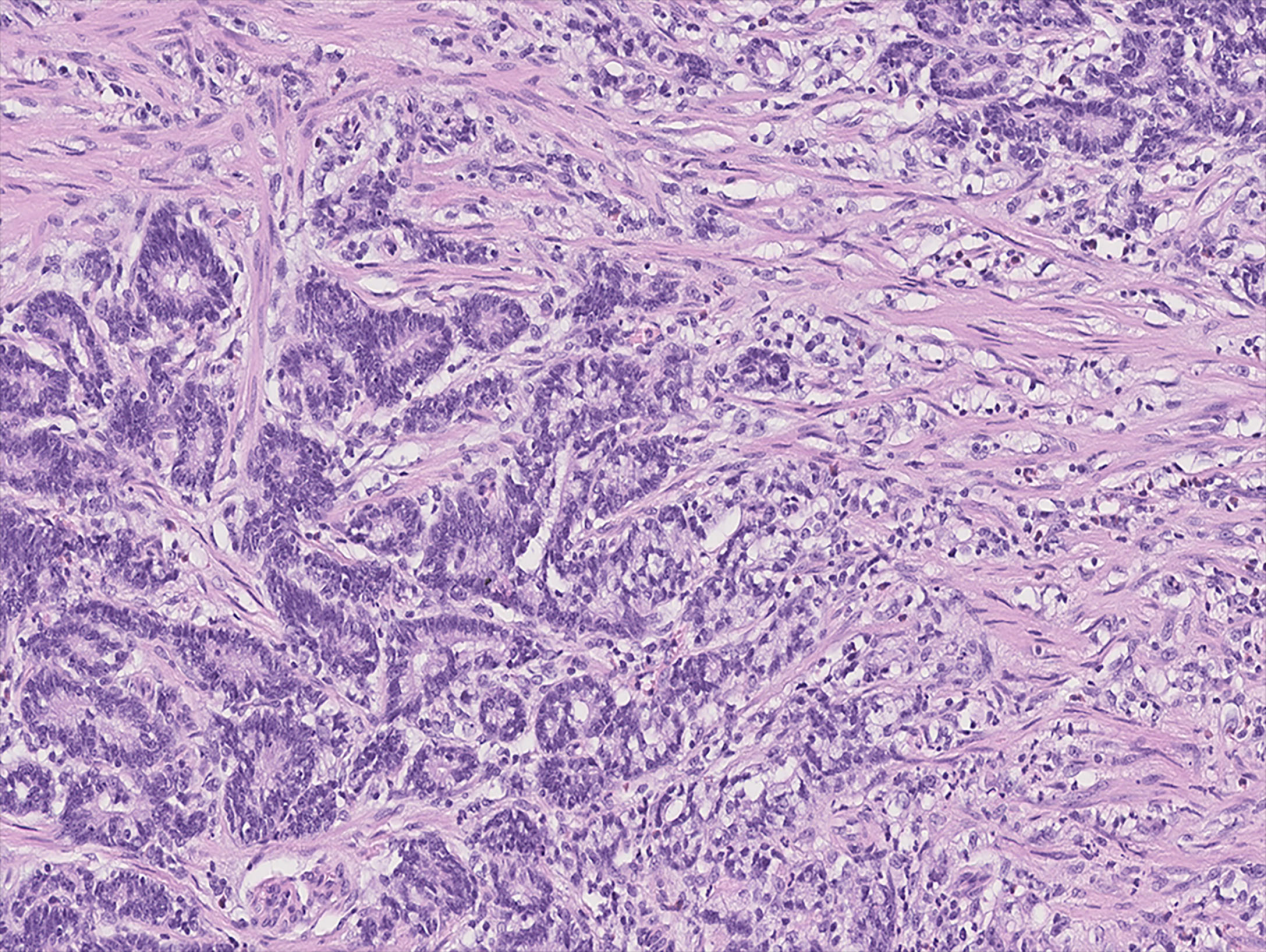
Photomicrograph of mixed-type gastric cancer with moderately differentiated tubular adenocarcinoma on the left and ≤10% SRC carcinoma on the right (Group 1).

**Figure 3:**
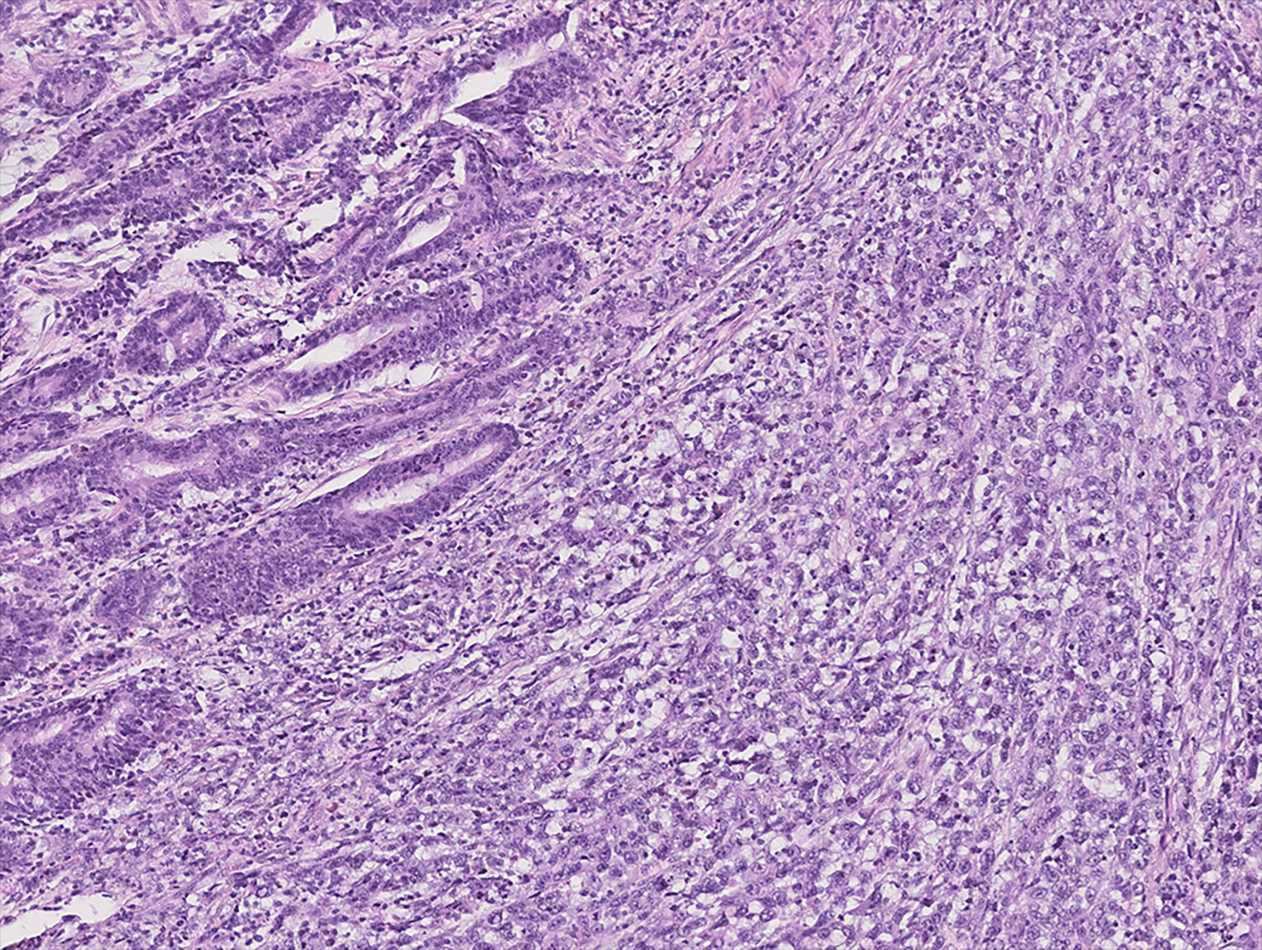
Photomicrograph of mixed-type gastric cancer with moderately differentiated tubular adenocarcinoma on the left and 10%–90% SRC carcinoma on the right (Group 2).

Ninety-eight (59.7%) patients were classified as “Group 1” (**Figure 2**), 66 (40.3%) patients were classified as “Group 2” (**Figure 3**), and none of the 164 patients were considered as “Group 3”. The maximum value of the SRC percentage was 40%. **Tables 1**, **2** also summarize the analysis of the clinicopathological and surgical characteristics of Group 1 and Group 2. A higher proportion of patients in Group 1 had a G3 grading (34.7% vs. 16.7%, *p* < 0.001) and almost none had a G1 grading (5.1% vs. 21.2%, *p* < 0.001). Group 2 had a lower mean tumor size (±SD) (42.5 ± 24.1 vs. 56.7 ± 26.4 mm, *p* < 0.001), and a lower proportion of Group 2 patients had AGC (69.7% vs. 94.9%, *p* < 0.001). The prevalence of SRC >10% was 80% (20/25) in ECG and 33.1% (46/139) in AGC patients. Additionally, Group 1 more frequently observed serosal invasion, positive peritoneal cytology, nodal involvement, neural and lymphovascular invasion, and R+ resection than Group 2. In Group 1, the median of positive nodes was 11 (range, 1–86) tumors compared to 1 (range, 1–54) in Group 2 (*p* < 0.001).

### Survival Analysis

One hundred twenty-three patients who received curative resection (R0) were suitable for survival analysis. The median follow-up was 153 months [95% CI 144.3–161.7]. The median OS time for the entire group of patients with mixed-type GC was 69 months [95% CI 36.2–101.8]. The 5-, 10-, and 15-year OS rates were 53.3%, 37.8%, and 24.6%, respectively. The median RFS for the whole cohort was 10 months [95% CI 7.9-12.1], with a 3-year RFS rate of 11.4%.

**Figure 4:**
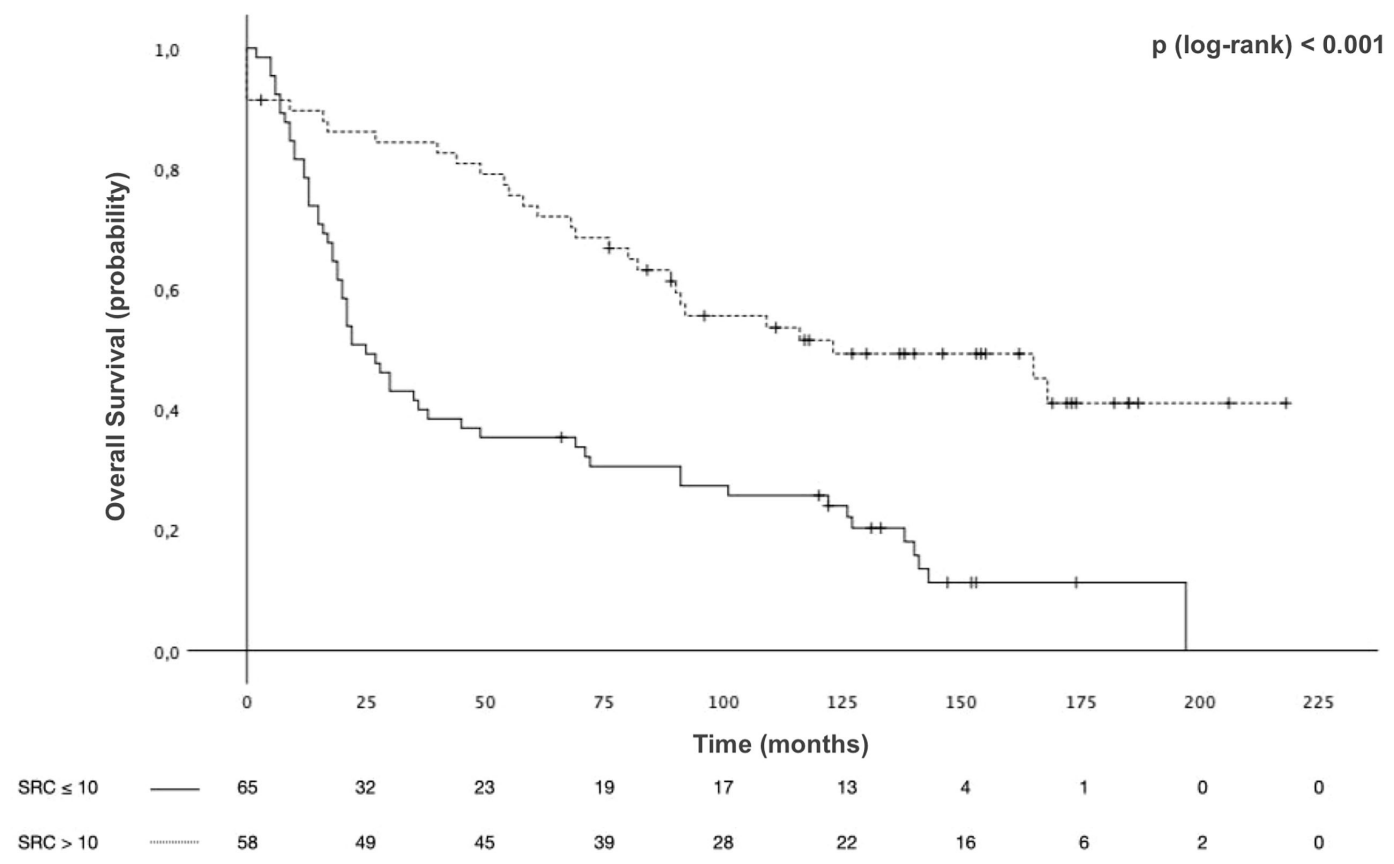
Overall survival (OS) of patients with mixed-type gastric cancers stratified by signet ring cell (SRC) proportion.

**Figure 5:**
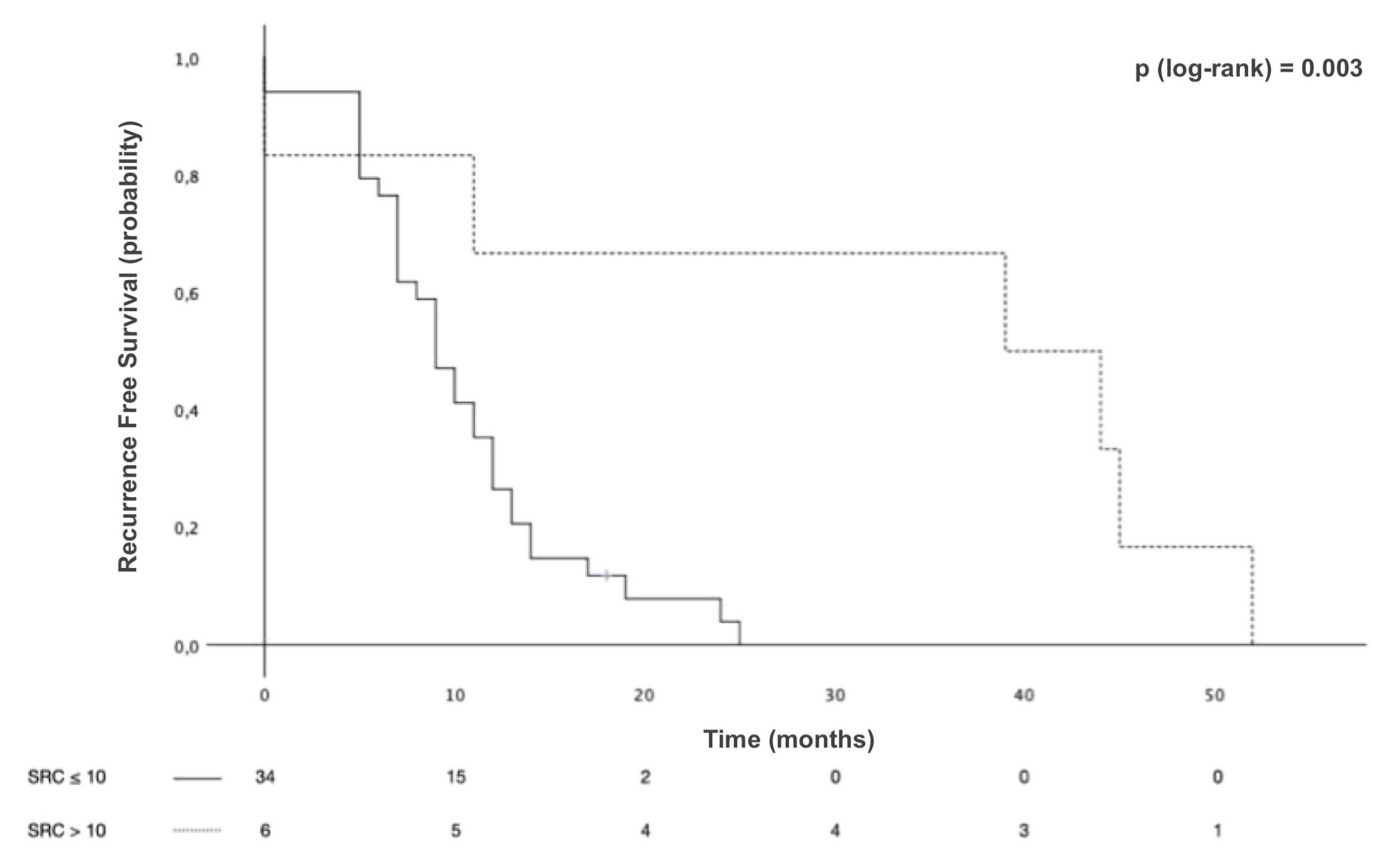
Recurrence-free survival (RFS) of patients with mixed-type gastric cancers stratified by signet ring cell (SRC) proportion.

Survival analyses were conducted with the mixed-type GCs grouped according to SRC proportion. The median OS in Group 1 was 25 months [95% CI 17.1–32.9] and that in Group 2 was 123 months [95% CI 52.8–193.2], with 5-year OS of 35.4% and 73.8% and 10-year OS of 25.7% and 51.5%, *p* < 0.001 (**Figure 4**). The median RFS in Group 1 was 9 months [95% CI 7.1–10.9] and 39 months [95% CI 0–78.6] in Group 2, with 3-year RFS of 0% and 66.7%, respectively, *p* = 0.03 (**Figure 5**).

Interestingly, Group 2 showed better median OS [168 months (95% CI 65.3–270.6)] among patients with EGC, compared to Group 1 [69 months, (95% CI 0–152.7)], *p* < 0.001. Results for AGC patients were similar, with a median OS of 123 months [95% CI 43.4-202.6] for Group 2, compared with a median OS of 22 months [95% CI 13.4-30.6] for Group 1, *p* < 0.001. Given the poor prognosis found in univariate analysis for these variables, a multivariable model was constructed to adjust for potential confounding factors, which is illustrated in **Table 3**. The increase in mortality risk was about threefold in patients with ≤10% SRC pattern compared to those with >10% [HR 2.70 (95% CI 1.72–4.24), *p* < 0.001]. After adjusting for potential confounding factors, the SRC percentage was still an independent predictor of survival. Lymph node metastasis served as another independent prognostic factor.

**Table 3:** Univariate analysis of factors affecting survival and hazard ratios for risk factors of mortality.

Variable	Category	*N*	Median overall survival (months) [95% CI]	Univariate analysis (*p* value)	Multivariate analysis		
					HR	95% CI	*p*-value
Gender	Male	83	58 [20.8–95.2]	0.552			
	Female	40	89 [63.8–114.2]				
Age	<60 years	12	140 [NR]	0.080			
	≥60 years	111	68 [41.6–94.4]				
Lymph node metastasis	Negative	72	116 [86.1–145.9]	**0.000**	1		
	Positive	51	21 [13.1–28.9]		2.1	[1.03–4.29]	**0.041**
Lymphovascular invasion	Negative	77	101 [68.1–133.9]	**0.000**	1		
	Positive	40	21 [17.3–24.7]		1.1	[0.52–2.20]	0.855
Perineural invasion	Negative	76	80 [50.4–109.6]	**0.006**	1		
	Positive	11	17 [8.7–25.3]		1.5	[0.62–3.46]	0.391
EGC/AGC	Early Gastric Cancer	25	116 [22.4–209.6]	**0.012**	1		
	Advanced Gastric Cancer	98	49 [16.2–81.8]		1.04	[0.43–2.47]	0.937
TNM stage	T1–2	60	109 [72.7–145.3]	**0.000**	1		
	T3–4	63	25 [10.4–39.6]		1.8	[0.88–3.54]	0.108
Adjuvant chemotherapy	Yes	95	118 [84.3–147.2]	**0.000**	1		
	No	69	23 [15.1–35.7]		2	[1.02–3.76]	**0.045**
Signet ring cells proportion	SRC ≤ 10	65	25 [17.1–32.9]	**0.000**	1.8	[1.04–3.19]	**0.035**
	SRC > 10	58	123 [52.8–193.2]		1		

Overall survival is illustrated as median with 95% CI. HR, hazard ratio; CI, confidence interval; NR, not reached; EGC, early gastric cancer; AGC, advanced gastric cancer; SRC, signet ring cell. P values in bold are statistically significant.

## Discussion

Our results showed that a high percentage of SRCs in mixed-type GC was linked to the most favorable clinicopathological characteristics as well as better survival outcomes, providing new insight into the evidence of the prognostic value of SRC also in mixed-type GC.

GCs exhibit a highly heterogeneous nature in terms of histological type and differentiation (1, 15). In the age of tailored surgery, it has been of greater interest not only to merely classify the tumor from a histological point of view but also to establish a pathological classification system with an independent prognostic peculiarity pursuing personalized clinical management. Although several classification systems for GC have been proposed over the years (3, 4, 16–21), the question of which of them may be relevant in the clinical setting has not yet been answered. Additionally, it would appear that the 50% SRC threshold proposed in the previous classifications has been vague. The World Health Organization (WHO) classification system is recognized as one of the most detailed among all classification systems and is employed by several researchers investigating the pathological and prognostic aspects of GC (1). Additionally, the 5th update of WHO classification has led to a marked improvement in GC knowledge. The mixed type has been described as a distinct histopathological entity that includes both glandular (tubular/papillary) and SRC/poorly cohesive components. Similarly, the Lauren classification, with a comparable significance to that provided by WHO, also identifies mixed type as a distinct category with a mixture of intestinal and diffuse components (4, 22). Evidence gathered over the last years has shown conflicting results, and the prognostic impact of this histotype is still debated among the scientific community, with some authors describing the mixed type as a predictor of poor prognosis and high risk of lymph node metastasis (23–25) and others reporting similar outcomes compared with other histotypes (26, 27).

Our group previously reported that the percentage of SRCs is inversely related to tumor aggressiveness in poorly cohesive GCs confirming the role of SRC pattern as an independent predictor of survival (8). By extending the concept of this new evidence to mixed histology, we can postulate that the inconsistent findings of previous studies (23–28) could be explained by the heterogeneity of SRC components. To the best of our knowledge, our study is the first to evaluate the clinicopathological aspects and prognostic outcomes of mixed-type GCs according to SRC percentages. Nonetheless, the retrospective design and the small sample size could affect our results in terms of bias.

As discussed, several studies showed that mixed histotype is associated with aggressive behavior, such as tumor size, lymphatic invasion, and lymph node metastasis (23, 29, 30). On the other hand, Zhong et al. (26), in a retrospective study on a total of 298 patients, stated that histological mixed type was not an independent risk factor for lymph node metastasis and was not associated with more aggressive characteristics in comparison with other histotypes. The same results were also obtained by Min et al. (27) on a cohort of 1577 patients.

In an attempt to explain the discrepancies arising from previous studies, this paper introduces new and intriguing evidence as regards the mixed-type GCs. By evaluating histopathological characteristics and prognoses, we observed that compared with those with more than 10% of SRCs, cancer patients with less than 10% of SRCs were younger and were more frequently associated with increased size, depth of invasion, nodal involvement, and advanced pathologic stage at diagnosis. Additionally, our data highlighted a distinctive tendency toward a survival difference according to SRC percentage between the two groups of mixed-type GC. Definitively, according to this research, the SRC pattern was an independent factor predicting prognosis in mixed-type GC patients.

Molecular mechanisms underlying the unfavorable behavior are still unclear. Anyway, it has been postulated that more aggressive clinicopathological as well as morphological findings of mixed-type cancers (10, 31) could be attributed to the angiogenetic process and cell proliferation, to cytosine-phosphate-guanine (CpG) island hypermethylation (32), or to the unregulated expression of proteins such as Ki-67, E-cadherin, and VEGF proteins, which were involved in proliferation (10), adhesion, and angiogenesis activities (10, 28). Interestingly, some researchers argued that mixed type would undergo transitional events leading to phenotype transformation along with cancer progression (33). Based on this finding, further research aiming at SRC amount comparison between endoscopic biopsies and resected specimens could be conducted.

We provide compelling evidence suggesting that distinct clinicopathological and prognostic findings are associated with the amount of SRCs in mixed-type gastric tumors. The significance of these different SRC percentages has not been investigated deeper, and our focus on the clinical outcome rather than genetic alterations could represent a limit for our study. Nevertheless, our data have confirmed, through undeniable evidence, that the SRC pattern is an independent predictor of survival also in mixed-type GC. Results demonstrate that a lower SRC proportion is associated with poorer OS. Thus, the proportion of SRCs can be considered a marker of differentiation.

In conclusion, the percentage of SRCs is inversely related to aggressive behavior and poor prognosis in mixed-type GCs, highlighting the role of SRC amount as an independent predictor of survival. Further studies are needed to investigate specific genetic and molecular profiles underlying the SRC effects to reach a clearer interpretation of these findings.

## Data Availability Statement

The raw data supporting the conclusions of this article will be made available by the authors, without undue reservation.

## Ethics Statement

The studies involving human participants were reviewed and approved by the Institutional Review Committee of the University of Siena. The patients/participants provided their written informed consent to participate in this study.

## Author Contributions

Study concepts: LMar, FR, MA, and KP. Study design: LMar, FR, OS, and KP. Data acquisition: LMar, MA, LR, LMal, and KP. Quality control of data and algorithms: VS, MC, LMal, RP, IB, and OS. Data analysis and interpretation: LMar, DM, and MA. Statistical analysis: LMar, LR, RP, and IB. Manuscript preparation: LMar, DM, FR, VS, LR, RP, MA, and MC. Manuscript editing: LR, LC, RP, MA, and OS. Manuscript review: LMar, MA, MC, VS, LR, LC, IB, and KP. All authors contributed to the article and approved the submitted version.

## Conflict of Interest

The authors declare that the research was conducted in the absence of any commercial or financial relationships that could be construed as a potential conflict of interest.

## Publisher’s Note

All claims expressed in this article are solely those of the authors and do not necessarily represent those of their affiliated organizations, or those of the publisher, the editors and the reviewers. Any product that may be evaluated in this article, or claim that may be made by its manufacturer, is not guaranteed or endorsed by the publisher.
